# Appendicitis on the Wrong Side: A Case of Left-Sided Acute Appendicitis With Intestinal Nonrotation

**DOI:** 10.7759/cureus.115045

**Published:** 2026-08-23

**Authors:** Swathi Mulupuru, Srinidhi Srinivasan, Arun Paul, Lakshmi Chakradhar Yarlagadda

**Affiliations:** 1 Emergency Medicine, Alluri Sitarama Raju Academy of Medical Sciences, Eluru, IND; 2 Radiology, Alluri Sitarama Raju Academy of Medical Sciences, Eluru, IND; 3 Internal Medicine, GSL Medical College, Rajahmundry, IND

**Keywords:** acute abdomen, case report, intestinal nonrotation, left-sided acute appendicitis, midgut malrotation, superior mesenteric artery

## Abstract

Left-sided acute appendicitis (LSAA) is a rare clinical entity, most commonly associated with midgut malrotation (MM) or situs viscerum inversus (SVI). Its atypical presentation poses a significant diagnostic challenge for emergency physicians, radiologists, and surgeons.

We report the case of a 53-year-old man who presented with a two-day history of left-sided abdominal pain, fever, and vomiting. Laboratory investigations revealed marked leukocytosis. Abdominal ultrasonography identified an appendix-like structure with surrounding inflammation in the left iliac fossa. Contrast-enhanced computed tomography (CT) of the abdomen confirmed midgut nonrotation, with the ascending colon positioned on the left side and an abnormal superior mesenteric artery-to-vein relationship. The patient underwent open appendectomy through a midline incision. Intraoperative findings confirmed LSAA with large bowel nonrotation. The postoperative course was complicated by peritonitis, which was managed with surgical intervention and broad-spectrum antibiotics. At the 21-day follow-up, the patient was asymptomatic.

This case highlights the importance of maintaining a high index of suspicion for LSAA in patients presenting with atypical left lower quadrant pain. Thorough preoperative imaging, particularly CT, is essential for accurate diagnosis and operative planning. Prompt surgical management remains the cornerstone of treatment to prevent life-threatening complications.

## Introduction

Acute appendicitis is the most common surgical cause of acute abdominal pain, with lifetime risks of 8.6% in males and 6.7% in females. Left-sided acute appendicitis (LSAA), characterized by an anatomical variation in the position of the appendix and atypical clinical features, typically occurs in association with two congenital anomalies: situs viscerum inversus (SVI) and midgut malrotation (MM) [1,2].

Intestinal malrotation results from abnormal rotation and fixation of the embryonic midgut around the axis of the superior mesenteric artery during fetal development. MM is predominantly diagnosed in childhood, although it may remain unrecognized until adulthood [3]. The anatomical spectrum includes nonrotation, incomplete rotation, and reverse rotation [4]. In intestinal nonrotation, the expected adult relationship between the small and large bowel is altered, with the small bowel predominantly positioned on the right and the large bowel on the left. Abnormal positioning of the caecum and appendix may therefore result in left-sided appendicitis and atypical localization of abdominal pain.

Some patients remain asymptomatic and are diagnosed incidentally or only when complications occur in adulthood. In adults, the atypical anatomical configuration may create diagnostic difficulties when symptoms do not correspond to the usual location of acute appendicitis. Cross-sectional imaging, particularly contrast-enhanced computed tomography (CT), can demonstrate abnormal bowel distribution and mesenteric vascular relationships and may therefore be important for establishing the diagnosis and planning surgical management.

LSAA associated with intestinal nonrotation is uncommon. We report the case of a 53-year-old man who presented with left iliac fossa pain and was diagnosed with left-sided acute appendicitis associated with intestinal nonrotation on imaging, with the diagnosis subsequently confirmed intraoperatively.

## Case presentation

A 53-year-old man presented with a two-day history of left-sided abdominal pain, occasional vomiting, and a single episode of fever. The pain was localized to the left iliac fossa, was sharp in nature, and was rated as 7/10 in intensity. He denied diarrhea or other gastrointestinal or urinary symptoms. His family history was unremarkable. On physical examination, his pulse rate was 88 beats/min, blood pressure was 130/90 mmHg, and respiratory rate was 22 breaths/min. He had pain and tenderness localized to the left iliac fossa, without guarding, rigidity, or rebound tenderness. Bowel sounds were decreased. Laboratory investigations revealed a leukocyte count of 19,400/µL, hemoglobin of 11.8 g/dL, packed cell volume (PCV) of 36.4%, and neutrophils of 93.6%. Renal function and serum electrolytes were within normal limits. C-reactive protein was not available.

**Figure 1:**
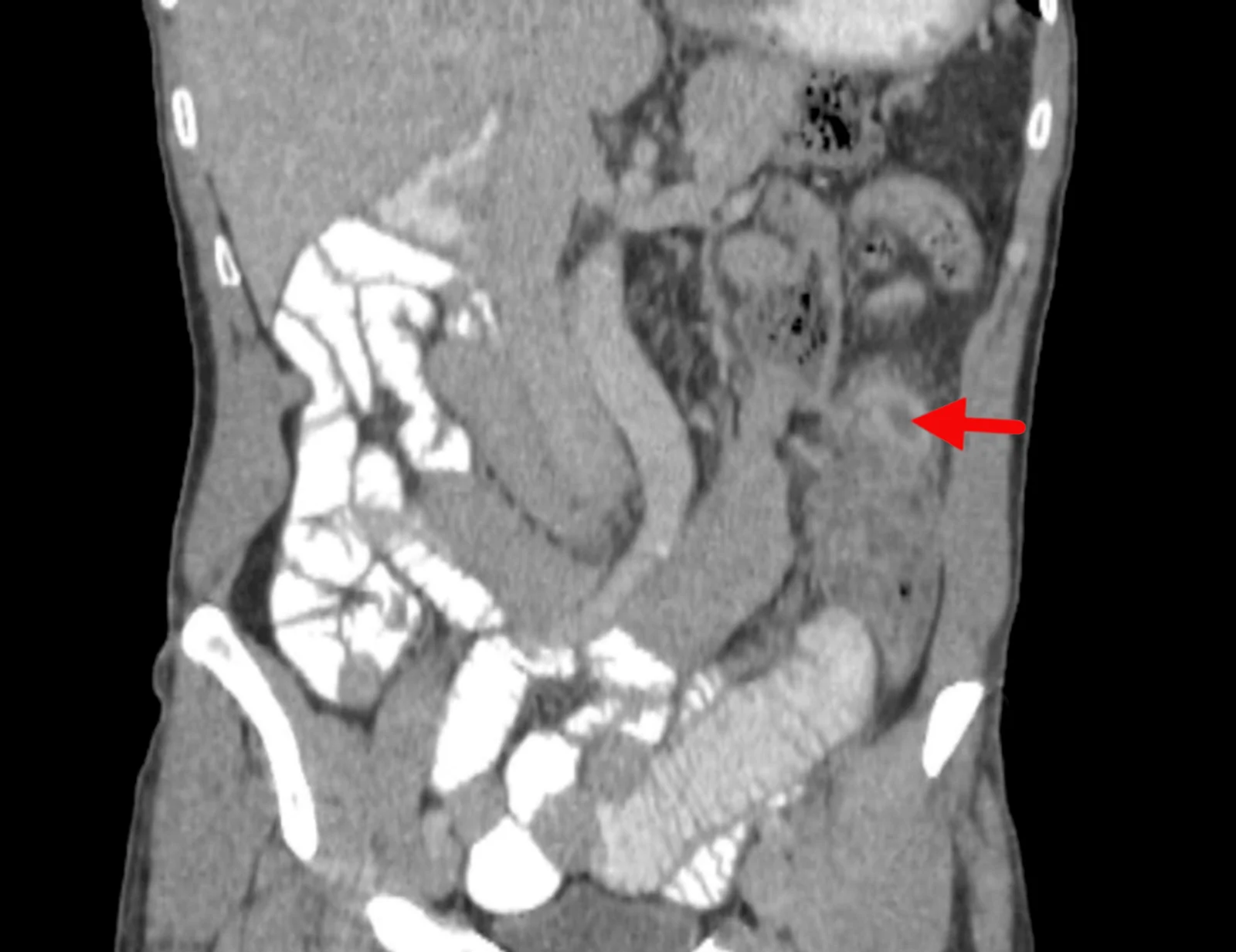
Left-sided appendicitis on coronal CT Coronal contrast-enhanced CT image demonstrating a thick-walled, dilated, inflamed appendix located in the left lower abdomen, consistent with left-sided acute appendicitis.

**Figure 2:**
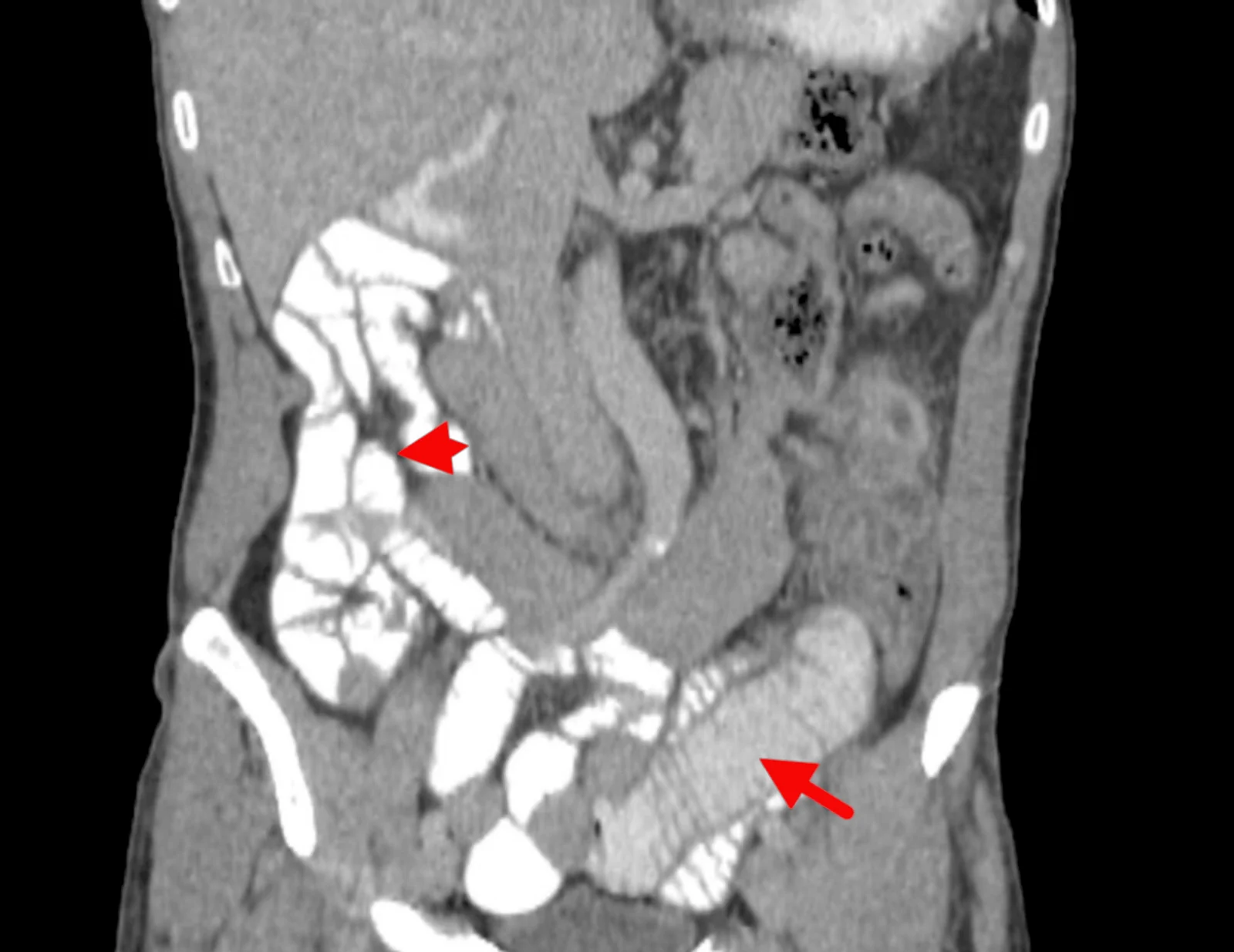
Intestinal nonrotation on coronal CT Coronal contrast-enhanced CT image demonstrating an abnormal bowel arrangement, with the small bowel loops (arrowhead) predominantly located in the right abdomen and the large bowel loops (arrow) predominantly located in the left abdomen, consistent with intestinal nonrotation.

**Figure 3:**
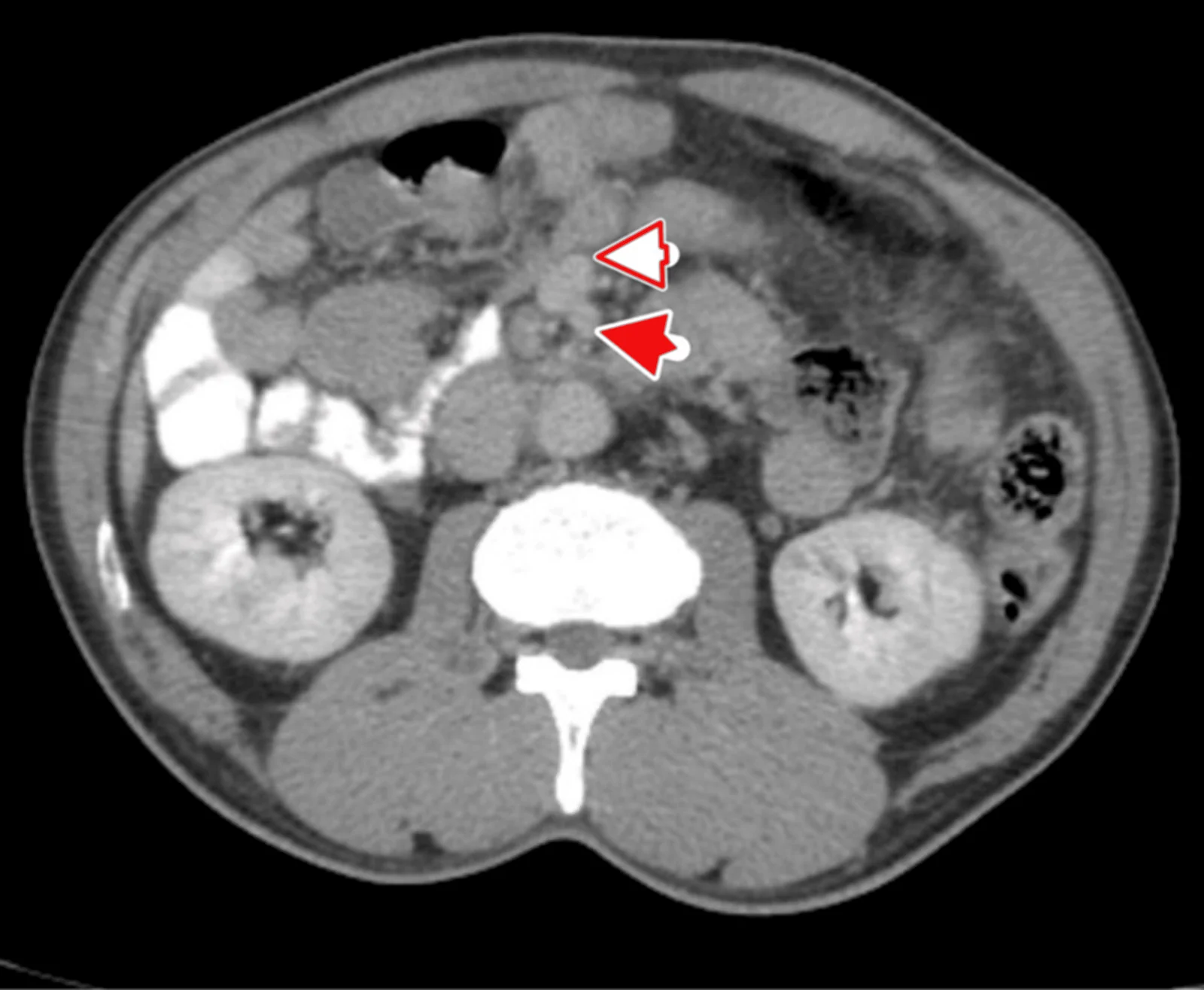
Intestinal nonrotation on axial CT Axial contrast-enhanced CT image demonstrating an inverse superior mesenteric artery (SMA)/superior mesenteric vein (SMV) relationship, with the SMV (arrowhead outline) located anterior to the SMA (arrowhead tapered), consistent with intestinal malrotation.

**Figure 4:**
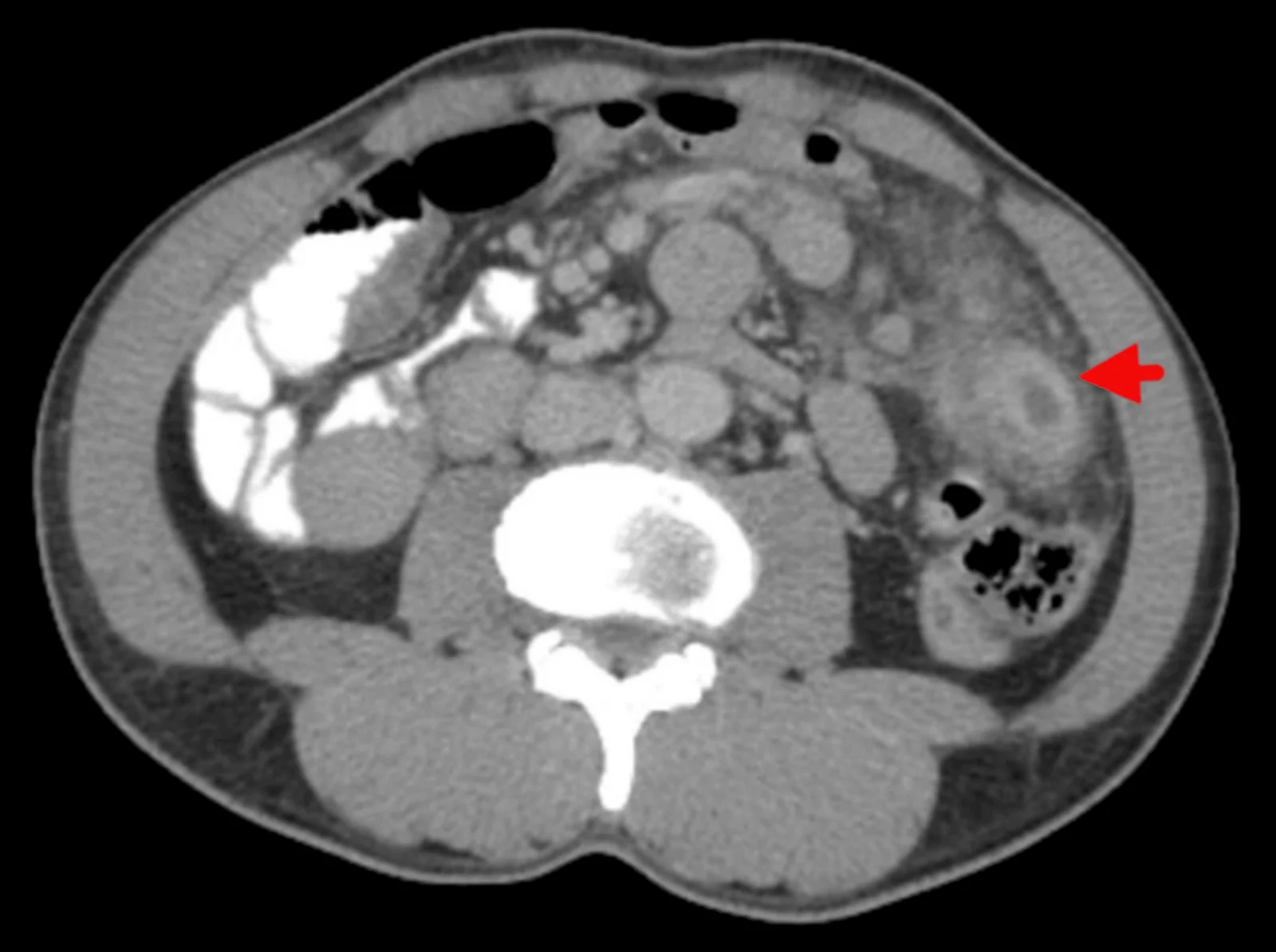
Acute appendicitis on axial CT Axial contrast-enhanced CT image demonstrating a left-sided dilated fluid-filled appendix, with surrounding inflammatory fat stranding, consistent with left-sided acute appendicitis.

CT of the abdomen demonstrated tenderness in the left iliac fossa and lumbar regions, along with an appendix-like structure showing features of inflammation and adjacent lymph nodes, suggestive of LSAA (Figure 1). Plain and contrast-enhanced CT scans demonstrated the ascending colon positioned on the left side and running parallel to the descending colon, while the small bowel was predominantly positioned on the right side (Figure 2). The CT scan also demonstrated the superior mesenteric vein anterior to the superior mesenteric artery, representing an abnormal superior mesenteric vessel relationship (Figure 3). Taken together with the abnormal bowel distribution, these findings were consistent with intestinal nonrotation associated with LSAA (Figure 4).

**Figure 5:**
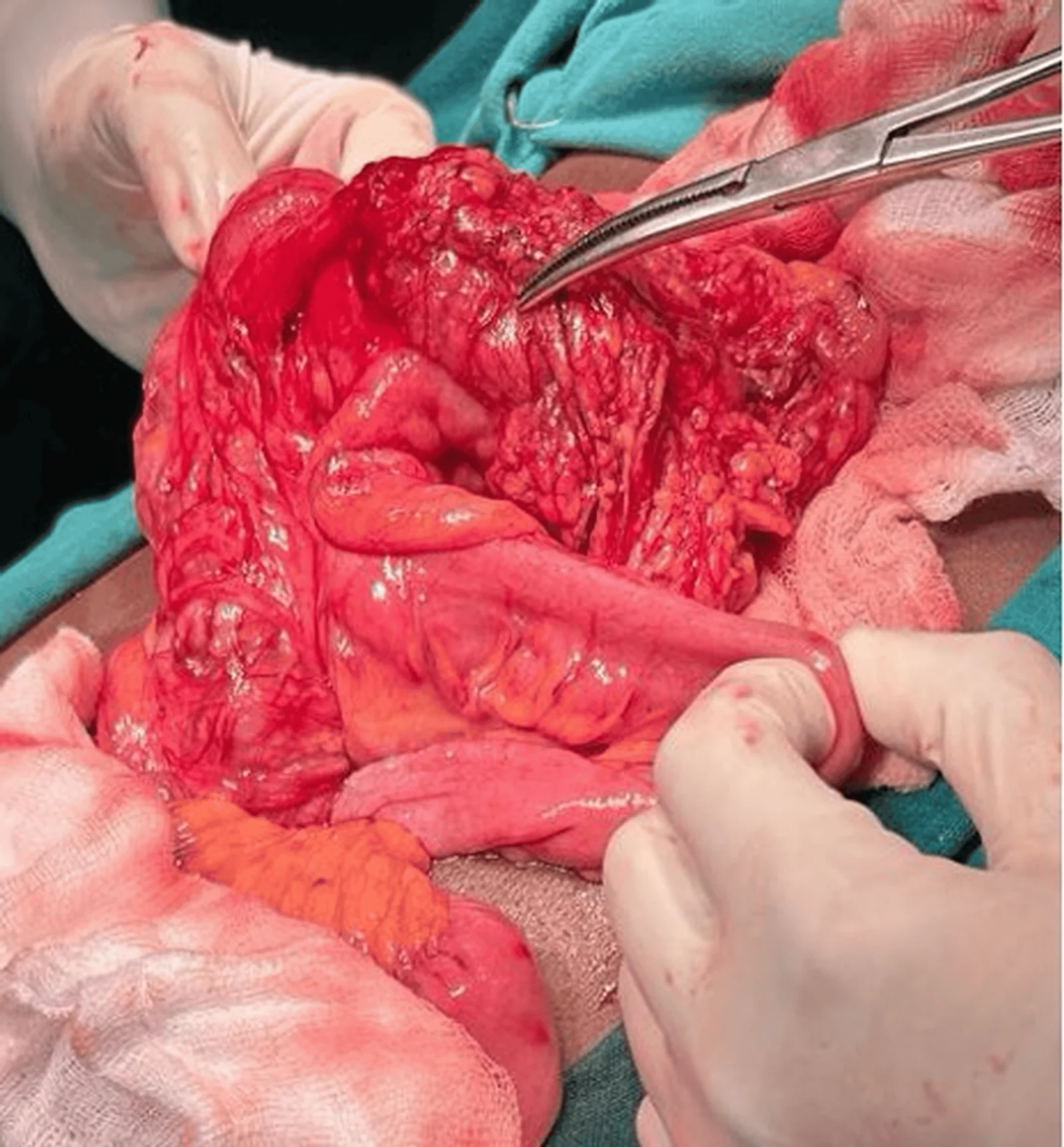
Intraoperative findings of left-sided acute appendicitis with intestinal nonrotation

**Figure 6:**
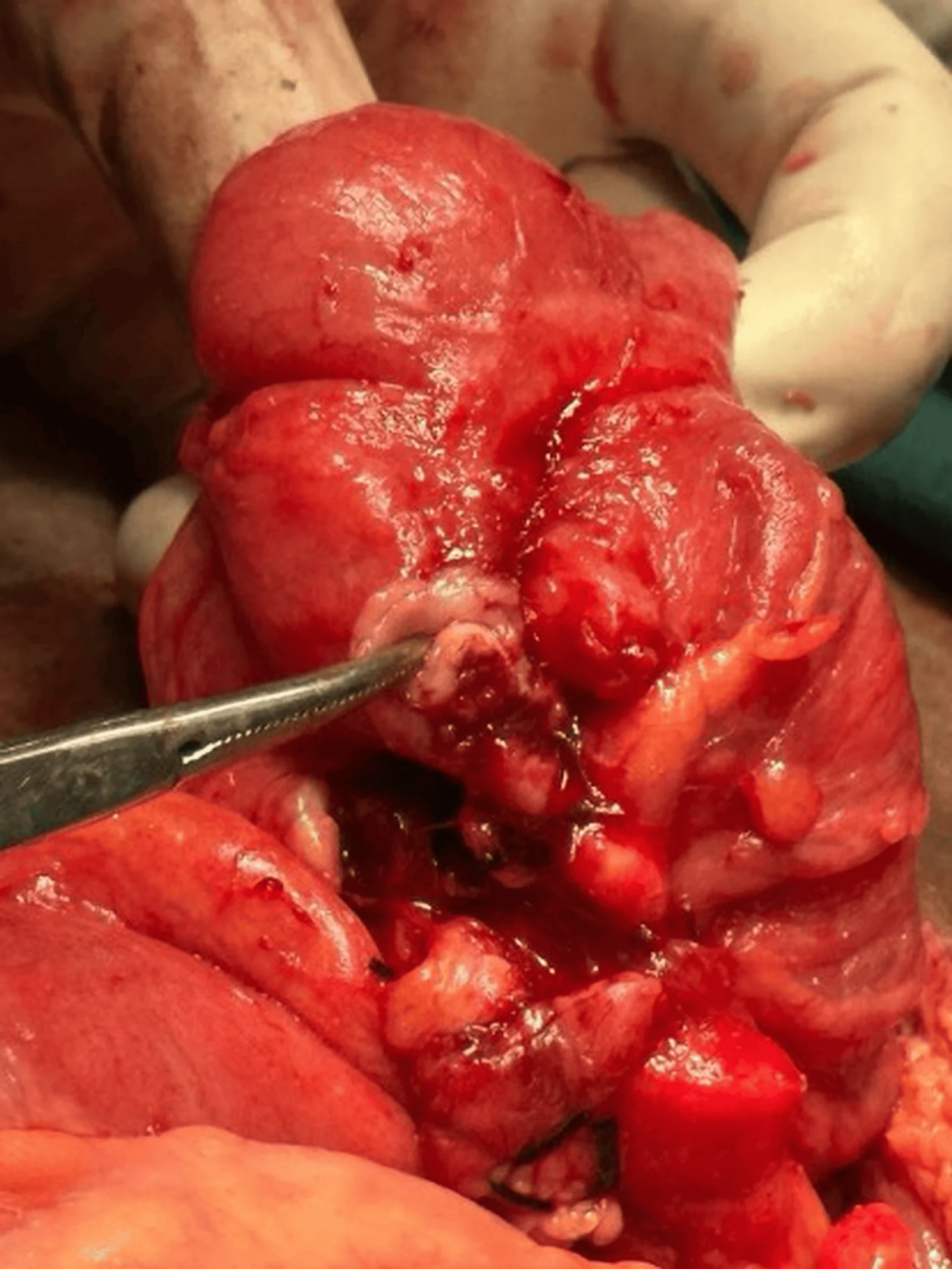
Appendicular stump after removal of the inflamed appendix

In the emergency department, the patient was administered Ringer's lactate and tramadol for fluid resuscitation and pain relief, respectively. Following review of the CT findings, surgical intervention was recommended. An appendectomy was performed through a midline incision. Intraoperatively, the appendix and cecum were identified in a left-sided position, consistent with intestinal nonrotation (Figure 5). No Ladd procedure was performed for the nonrotation because of the perceived low risk of intestinal obstruction. Histopathological examination of the resected specimen confirmed acute suppurative appendicitis with periappendiceal inflammation. No evidence of malignancy was identified. On postoperative day 3, the patient developed peritonitis, presenting with abdominal pain, guarding, and rigidity. Tachycardia was also noted. A repeat contrast-enhanced CT scan of the abdomen revealed an intra-abdominal collection suggestive of a leak from the appendiceal stump (Figure 6). In view of the clinical and radiological findings, the patient was diagnosed with postoperative peritonitis and underwent re-exploration, thorough peritoneal lavage, and drainage. Broad-spectrum antibiotic therapy with piperacillin-tazobactam (4.5 g) and metronidazole (500 mg), both administered three times daily, was initiated. At the 21-day follow-up, the patient was asymptomatic.

## Discussion

Acute appendicitis usually presents with vague epigastric or periumbilical pain that subsequently migrates to the right lower quadrant, accompanied by anorexia, nausea, vomiting, and low-grade fever. Malrotation anomalies (MM) occur in approximately 0.03%-0.5% of live births [5]. MM results from failure of the primitive intestinal loop to rotate normally around the superior mesenteric artery during fetal development. It typically presents during the first month of life with bowel dysfunction and bilious vomiting; however, many cases, including those with symptomatic variant intestinal torsion (SVIT), may remain asymptomatic.

LSAA may present with atypical symptoms and anatomical findings, such as a right-sided appendix extending into the left lower quadrant. In a study of 71,000 cases of appendicitis, Collins reported an incidence of 0.04% for true LSAA, with 0.024% associated with MM and 0.016% with SVIT [6,7]. Akbulut et al. reported that among patients with LSAA, 62.1% presented with left lower quadrant pain, 14.7% with right lower quadrant pain, 7.3% with bilateral pain, 7.3% with left upper quadrant pain, 6.3% with periumbilical pain, and 2% with pelvic pain [8].

The differential diagnoses for left lower quadrant pain include colitis, diverticulitis, inflammatory bowel disease, intestinal obstruction (such as volvulus), nephrolithiasis, pyelonephritis, testicular torsion, epididymitis, atypical right-sided appendicitis, left-sided appendicitis, and primary epiploic appendagitis. LSAA associated with intestinal malrotation may be misdiagnosed as diverticulitis, potentially resulting in delays in appropriate management. In one reported case, such a misdiagnosis resulted in an emergency laparotomy [5,9,10]. Accurate and timely diagnosis is therefore essential to prevent complications.

Imaging is essential for accurate diagnosis and for confirming the presence of SVIT or MM. The increased use of imaging for acute abdominal pain has led to more frequent incidental identification of MM [11]. In the present case, the patient had no history of chronic abdominal pain and presented for the first time with acute symptoms. Preoperative diagnosis of LSAA can be challenging and requires a high index of suspicion. Ultrasonography and CT are useful modalities for diagnosing intestinal malrotation. Key imaging features of MM include the absence of a retroperitoneal duodenum, a medially and inferiorly positioned duodenojejunal junction, and an abnormal relationship between the superior mesenteric artery (SMA) and superior mesenteric vein (SMV), along with a left-sided large bowel and predominantly right-sided small bowel [12]. In the present case, the SMV was positioned anterior to the SMA, whereas under normal anatomical conditions in adults, the SMV lies to the right of the SMA.

Delayed diagnosis of appendicitis can lead to perforation and abscess formation. The atypical presentation associated with intestinal malrotation can further complicate diagnosis; however, appropriate imaging is crucial for accurate identification of LSAA and associated anatomical anomalies such as SVIT or MM. As in typical cases of appendicitis, appendectomy specimens from patients with LSAA should be submitted for histopathological examination. In the literature, 2 of 95 patients (a 59-year-old man and a 76-year-old woman) who underwent appendectomy for LSAA were found to have malignancy on histopathological examination [13,14]. Both patients subsequently required ascending hemicolectomy after pathology revealed mucinous adenocarcinoma and mucinous cystadenocarcinoma, respectively. In the present case, histopathological examination confirmed acute left-sided appendicitis with periappendiceal inflammation. The postoperative course was complicated by peritonitis following open appendectomy.

## Conclusions

LSAA is a rare condition that poses a diagnostic challenge because of its nonspecific and atypical clinical presentation, which may lead to delayed diagnosis and treatment. Greater awareness among emergency physicians, radiologists, and surgeons is essential to facilitate timely diagnosis and intervention. Appropriate imaging, along with laparoscopy when indicated, is critical for distinguishing LSAA from other causes of left lower quadrant pain and for identifying associated anatomical anomalies such as SVIT or MM. Early and appropriate surgical management, including both open and laparoscopic appendectomy, is vital to prevent complications such as perforation, abscess formation, and prolonged hospitalization.
